# Global knowledge gaps in acute febrile illness etiologic investigations: A scoping review

**DOI:** 10.1371/journal.pntd.0007792

**Published:** 2019-11-15

**Authors:** Chulwoo Rhee, Grishma A. Kharod, Nicolas Schaad, Nathan W. Furukawa, Neil M. Vora, David D. Blaney, John A. Crump, Kevin R. Clarke

**Affiliations:** 1 Division of Global Health Protection, Center for Global Health, Centers for Disease Control and Prevention, Atlanta, Georgia, United States of America; 2 Division of High-Consequence Pathogens and Pathology, National Center for Emerging and Zoonotic Infectious Disease, Centers for Disease Control and Prevention, Atlanta, Georgia, United States of America; 3 Department of Medicine, University of Washington, Seattle, Washington, United States of America; 4 Division of Infectious Diseases and International Health, Duke University Medical Center, Durham, North Carolina, United States of America; 5 Centre for International Health, University of Otago, New Zealand; The University of Sheffield, UNITED KINGDOM

## Abstract

**Background:**

Acute febrile illness (AFI), a common reason for people seeking medical care globally, represents a spectrum of infectious disease etiologies with important variations geographically and by population. There is no standardized approach to conducting AFI etiologic investigations, limiting interpretation of data in a global context. We conducted a scoping review to characterize current AFI research methodologies, identify global research gaps, and provide methodological research standardization recommendations.

**Methodology/Findings:**

Using pre-defined terms, we searched Medline, Embase, and Global Health, for publications from January 1, 2005–December 31, 2017. Publications cited in previously published systematic reviews and an online study repository of non-malarial febrile illness etiologies were also included. We screened abstracts for publications reporting on human infectious disease, aimed at determining AFI etiology using laboratory diagnostics. One-hundred ninety publications underwent full-text review, using a standardized tool to collect data on study characteristics, methodology, and laboratory diagnostics. AFI case definitions between publications varied: use of self-reported fever as part of case definitions (28%, 53/190), fever cut-off value (38·0°C most commonly used: 45%, 85/190), and fever measurement site (axillary most commonly used: 19%, 36/190). Eighty-nine publications (47%) did not include exclusion criteria, and inclusion criteria in 13% (24/190) of publications did not include age group. No publications included study settings in Southern Africa, Micronesia & Polynesia, or Central Asia. We summarized standardized reporting practices, specific to AFI etiologic investigations that would increase inter-study comparability.

**Conclusions:**

Wider implementation of standardized AFI reporting methods, with multi-pathogen disease detection, could improve comparability of study findings, knowledge of the range of AFI etiologies, and their contributions to the global AFI burden. These steps can guide resource allocation, strengthen outbreak detection and response, target prevention efforts, and improve clinical care, especially in resource-limited settings where disease control often relies on empiric treatment. PROSPERO: CRD42016035666.

## Author summary

Acute febrile illness (AFI) is a common reason for people seeking medical care globally with potentially serious infectious etiologies. However, AFI has no current consensus standardized approach when considered as a syndromic case definition for public health surveillance or research, especially in global settings where AFI treatment is performed with limited diagnostic availability. Therefore, the aim of this review was to describe current methodologies in AFI research, identify gaps in research, and provide recommendations for standardization of AFI research. We screened abstracts and completed full-text reviews on publications found through an extensive search. A total of 190 publications were included in the final review, from which we collected data on study characteristics, methodology, and laboratory diagnostics. These collected data elements allowed us to identify where there were inconsistencies in reporting and investigative methods, which data elements were not regularly collected, and what laboratory testing methods were commonly used. Standardized reporting methods for AFI investigations, along with laboratory testing capacity for multiple pathogens, can improve our knowledge of the causative agents of AFI in certain regions of the world. This can help us determine where resources are needed, how to strengthen outbreak detection and response, and how to improve medical care, especially in regions with limited resources.

## Introduction

Acute febrile illness (AFI) is often non-specific and used differently in clinical and public health settings. AFI in the clinical setting may simply refer to any illness with a fever. In public health surveillance and research, AFI has been frequently characterized as fever without an apparent localized site or cause of infection–a commonly occurring subset of febrile illnesses where empiric treatment guidance is needed. [1] Etiologic AFI investigations are valuable public health data sources. They contribute necessary information on disease prevalence, and help to inform estimates of morbidity, mortality, and economic impact. Such investigations are useful for guiding appropriate empiric treatment and case management, determining which resources need to be prioritized and where they need to be allocated, developing prevention and control measures, and detecting novel and emerging pathogens and outbreaks especially in areas where access to reliable confirmatory laboratory diagnostics for local causes of AFI is otherwise limited. Improved access to malaria diagnostics has generated much research interest in non-malaria causes of fever especially in countries with declining malaria incidence. [2–4]

However, there are challenges associated with current AFI investigations. Variability in AFI case definition use, inclusion and exclusion criteria for study enrollment, level of health system studied, absence of a control group to calculate attributable fractions, and poor comparability of diagnostic assays used contribute to the lack of standardized methodology for conducting AFI investigations, hampering the ability to compare findings, trends, and proportional etiology between studies and geographic locations. [5] Additionally, despite considerable heterogeneity of AFI etiology by population, region, and in time, there is limited published literature detailing these findings, both by geographic location and time period. [6] An absence of evidence-based AFI etiology data may result in unintended public health consequences when using necessary empiric syndromic case management. Over-use of antimicrobials and unnecessary antimalarial treatment have been reported to be common in low- and middle-income countries; [7] additionally, under-treatment can be an issue in severe diseases. Existing review articles aiming to summarize frequent pathogens responsible for causing AFI have emphasized the need for a more standardized methodological approach. [6–8]

The World Health Organization’s informal consultation on fever management in peripheral health care settings has called for a broad range of etiologic studies to identify the pathogens responsible for AFI. [9] Unlike influenza-like illness (ILI) or severe acute respiratory infection (SARI) where case definitions have been standardized globally, methodological standards for investigating AFI have not been generated. [10,11] With growing public health interest to apply AFI etiology data towards shared global health security goals, we conducted a scoping review to characterize recent AFI etiologic investigation methodologies and identify gaps useful for future public health action. Furthermore, this review summarizes commonly investigated pathogen-specific diagnostic testing utilized in AFI etiology research and provide comprehensive recommendations for standardized approaches to study methodology, reporting of AFI etiologic investigations, and associated recommended laboratory diagnostics.

## Methods

### Study design and literature search

A scoping review of existing literature is used primarily as a means of summarizing evidence to understand the extent and depth of a particular topic. [12–14] This method differs from a systematic review because it does not involve a quality assessment of studies. The Ovid platform was used to perform a comprehensive search through Medline, Embase, and Global Health, on English-language literature with publication dates from January 1, 2005–December 31, 2017. Search terms included “acute febrile illness,” “undifferentiated fever,” and “non-specific fever,” with Boolean operators (S1 Table). Articles cited in references from previously published systematic reviews and an online repository of studies on etiologies of non-malarial febrile illness were also reviewed (S1 Table). This scoping review was registered with PROSPERO international prospective register of systematic reviews (PROSPERO ID: CRD42016035666, University of York).

### Inclusion and exclusion criteria

**Fig 1:**
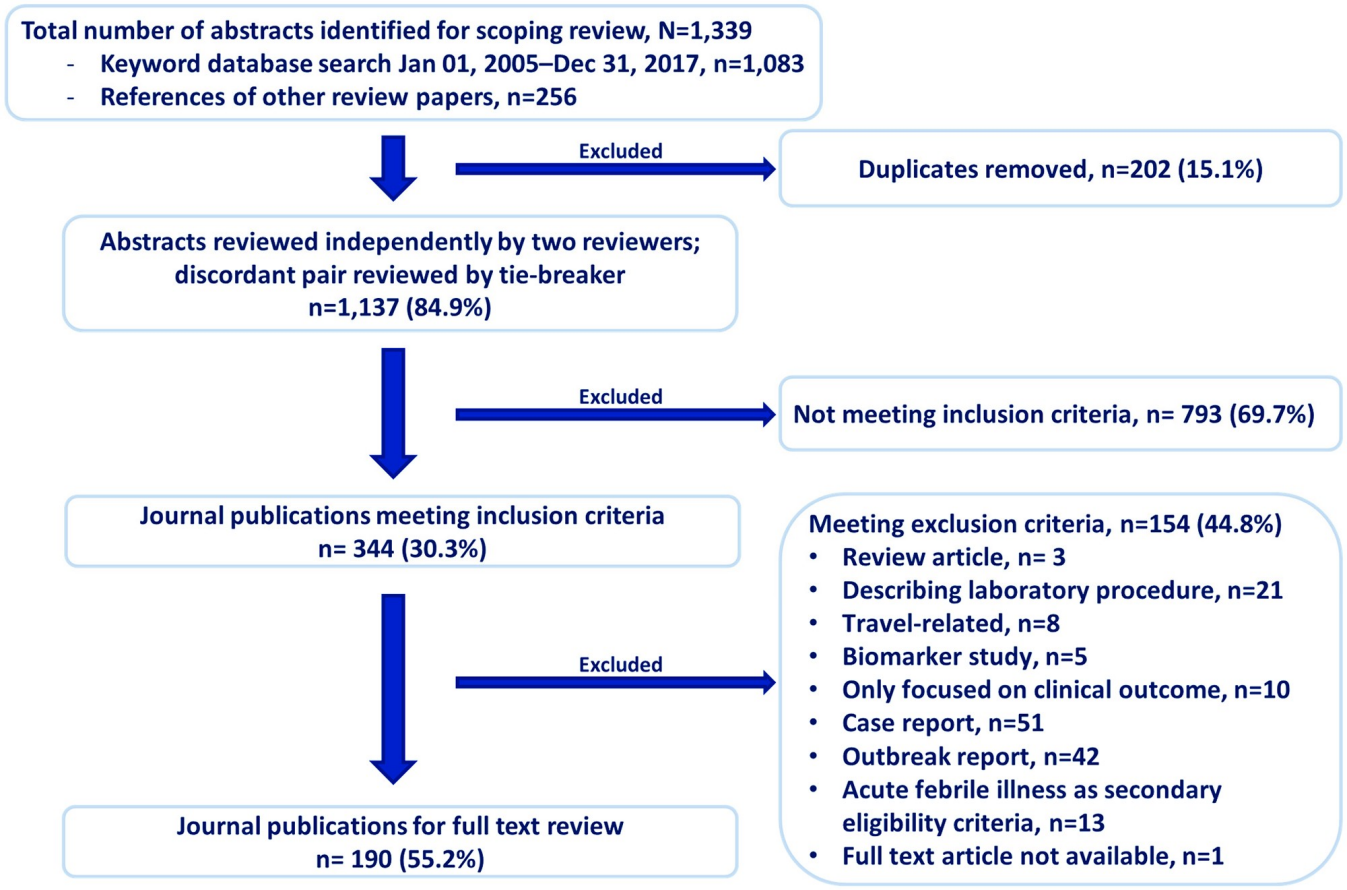
Literature search and abstract screening process used to determine the eligibility of publications on etiologies of acute febrile illness, published from January 01, 2005 to December 31, 2017 (N = 190).

Publications identified from the literature search were screened as part of a title and abstract review process to determine which ones would continue to the full-text review (Fig 1). For inclusion, the publications must have addressed human infectious disease, and aimed to determine AFI etiology using laboratory diagnostics. A publication was excluded if it was a review article without primary data, focused on the assessment of laboratory procedure or methods, report of travel-associated illness, biomarker study, report on clinical outcome only, case report, outbreak report, or a publication where AFI etiology determination was not the primary study goal (Fig 1). No geographic inclusion or exclusion criteria were applied. Two authors (CR, GK) independently reviewed each title and abstract to determine whether it should be included or excluded from further full-text review. For publications on which the initial review produced discordant conclusions, a third author (KC) independently performed a tiebreaker review. All publications that passed the title and abstract screening process were included in the full-text review.

### Full-text review and data analysis

The data characterization and collection form for the full-text review was created using Epi Info (Version 7·0, Centers for Disease Control and Prevention, Atlanta, GA, USA), and made available to all full-text reviewers through a web survey platform. Prior to data abstraction, specific instructions were provided, and publications were randomly assigned to reviewers.

Data collection included three primary areas: study characteristics, study methodology, and laboratory diagnostic methods (S1 File). Study characteristics included geographic location where the study was conducted, number of months during which participants were enrolled, and healthcare settings where participants were recruited. Countries were categorized following the United Nations regional and sub-regional classification. [15] Information on inclusion and exclusion criteria that were used in each publication were collected, with emphasis on AFI case definitions. For each pathogen, information on the type of specimen collected, laboratory diagnostic method used, and number of positives was gathered. Additional data abstraction on laboratory diagnostic methods was performed on commonly investigated pathogens defined as those appearing in more than ten publications, except for pathogens that were primarily detected through blood culture methods. This included an in-depth assessment of whether diagnostic methods used for pathogen detection were standard laboratory diagnostics and met laboratory confirmation case definition guidelines set forth by the CDC/Council of State and Territorial Epidemiologists (CSTE) or World Health Organization (WHO) (S2 Table). All analyses and data visualization for this scoping review were performed using Epi Info version 7·0. A final quality check was performed on the complete dataset to ensure data completeness and accuracy.

## Results

A total of 1,339 publications were initially identified for the scoping review; of these, 1,083 were identified through the keyword search and 256 were identified through references of review papers and the online repository of publications (S1 Table). After removal of 202 duplicate publications, and the title and abstract screening process that resulted in the removal of 793 publications that did not meet inclusion criteria and 154 publications that met exclusion criteria, 190 unique publications were included for full-text review and subsequent data analysis (Fig 1).

**Fig 2:**
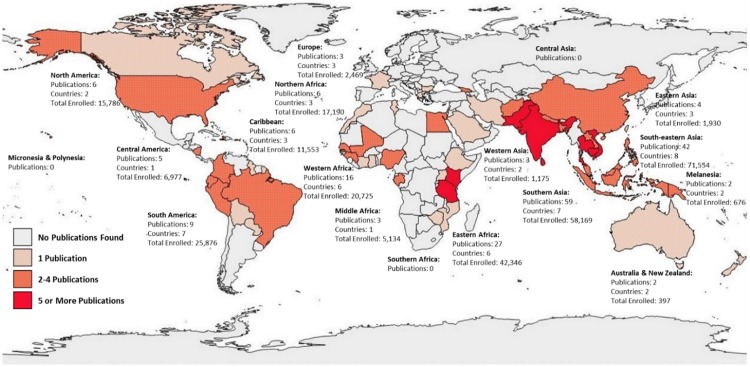
Geographic distribution by study location and number of study participants of publications on etiologies of acute febrile illness published from January 01, 2005 to December 31, 2017 (N = 190). (Source: Created specifically for this manuscript, using Epi Info 7; shape files from: https://www.naturalearthdata.com/downloads/10m-cultural-vectors/; data abstracted from full-text review process).

The geographic study setting with the most publications on AFI etiology was Southern Asia with 59 publications and 58,169 study participants; Southeastern Asia had the second highest number of publications with 42 publications and 71,554 study participants (Fig 2). There were no publications with study settings in Southern Africa, Micronesia & Polynesia, or Central Asia. Five (3%) of 190 reviewed publications had study data collection sites in multiple countries, while 25 (13%) included multiple sites within a single country; 160 (84%) had a single site in one country (Table 1).

**Table 1:** Data collection duration and characteristics of study sites reported by publications on etiologies of acute febrile illness published from January 01, 2005 to December 31, 2017 (N = 190).

Variables	Number of publications (%)
**Data collection duration**	
<12 months	47 (25%)
12–23 months	78 (41%)
24–47 months	47 (25%)
≥48 months	17 (8%)
Not reported	1 (1%)
**Reported geographic distribution of study sites**	
Single site in a country	160 (84%)
Multiple sites in a country	25 (13%)
Multiple sites in more than one country	5 (3%)
**Reported geographic setting of study sites**	
Urban	24 (13%)
Rural	29 (15%)
Both urban and rural	50 (26%)
Not reported	87 (46%)
**Reported healthcare setting of data collection sites**	
Inpatient only	58 (30%)
Outpatient only (facility-based)	21 (11%)
Community (non-facility-based)	5 (3%)
Inpatient and outpatient	64 (34%)
Not reported	42 (22%)

Of 189 publications that reported on duration of study data collection, 125 (66%) had data collection duration <24 months, 47 (25%) had a data collection duration of 24–47 months, and 17 (8%) publications had a data collection duration ≥48 months. In 64 (34%) publications, the study population consisted of both inpatient and outpatient study enrollees; 21 (11%) involved study populations consisting of only facility-based outpatient participants, while 58 (30%) consisted of only inpatient enrollees. Five (3%) involved study populations in community (non-facility-based) settings (Table 1). Healthcare settings for study populations were not reported in 42 (22%) of the 190 publications.

There was great variation in exclusion criteria, inclusion criteria, and AFI case definition between publications (Table 2). Exclusion criteria were not reported in 101 (53%) publications. Fifty-three (28%) publications reported inclusion of subjective or self-reported fever as part of their inclusion criteria. Sixty-six (35%) publications specifically reported a temperature measurement site, with axillary being the most commonly reported in 36 publications (19%). Measured temperature criteria for fever were described in 120 (63%) publications, with 38.0°C the most common value, in 85 (45%) of publications. In terms of age group, neonates (<1 month of age) were represented in 36 (19%) publications, and children 1–23 months of age were represented in 82 (43%) publications. In 24 (13%) publications, age group was not reported as part of the inclusion criteria or elsewhere within the publication. Thirteen (7%) publications reported on studies that involved enrollment of a non-febrile control group.

**Table 2:** Inclusion and exclusion criteria used to enroll study participants in studies on etiologies of acute febrile illness published from January 01, 2005 to December 31, 2017 (N = 190).

Reported Inclusion and Exclusion Criteria	Number of publications (%)
**Criteria for fever**	
Inclusion of subjective/self-reported fever	53 (28%)
Temperature measurement site specified*	66 (35%)
Axillary	36 (19%)
Tympanic	17 (9%)
Oral	16 (8%)
Rectal	8 (4%)
Temporal	1 (1%)
Fever cut-off value specified	120 (63%)
<37·5 °C	3 (2%)
37·5 °C	16 (8%)
37·8 °C	8 (4%)
38·0 °C	85 (45%)
38·3 °C	3 (2%)
≥38·5 °C	5 (3%)
**Inclusion of age group**†*	
<1 month	36 (19%)
1–23 months	82 (43%)
2–9 years	121 (64%)
10–19 years	150 (79%)
20–39 years	136 (72%)
40–59 years	133 (70%)
≥60 years	127 (67%)
Age for inclusion criteria not reported	24 (13%)
Exclusion criteria*	
Respiratory symptoms	23 (12%)
Malaria diagnosis	19 (10%)
No apparent focus of infection	19 (10%)
Gastrointestinal symptoms	15 (8%)
Urinary tract infection	14 (7%)
Malignancy diagnosis	13 (7%)
HIV diagnosis	10 (5%)
Skin infection or cellulitis	9 (5%)
Trauma or injury	8 (4%)
Exclusion criteria not described	101 (53%)
**Enrollment of control group**	13 (7%)

* Reported criteria are not mutually exclusive

^†^ In cases where age groups in the study crossed more than one age group category in Table 2, each age group category represented in the study was counted. For example, if the study participants were 2≤x≤15 years of age, the study was counted under each of the following categories in the table: 2–9 years, 10–19 years.

**Fig 3:**
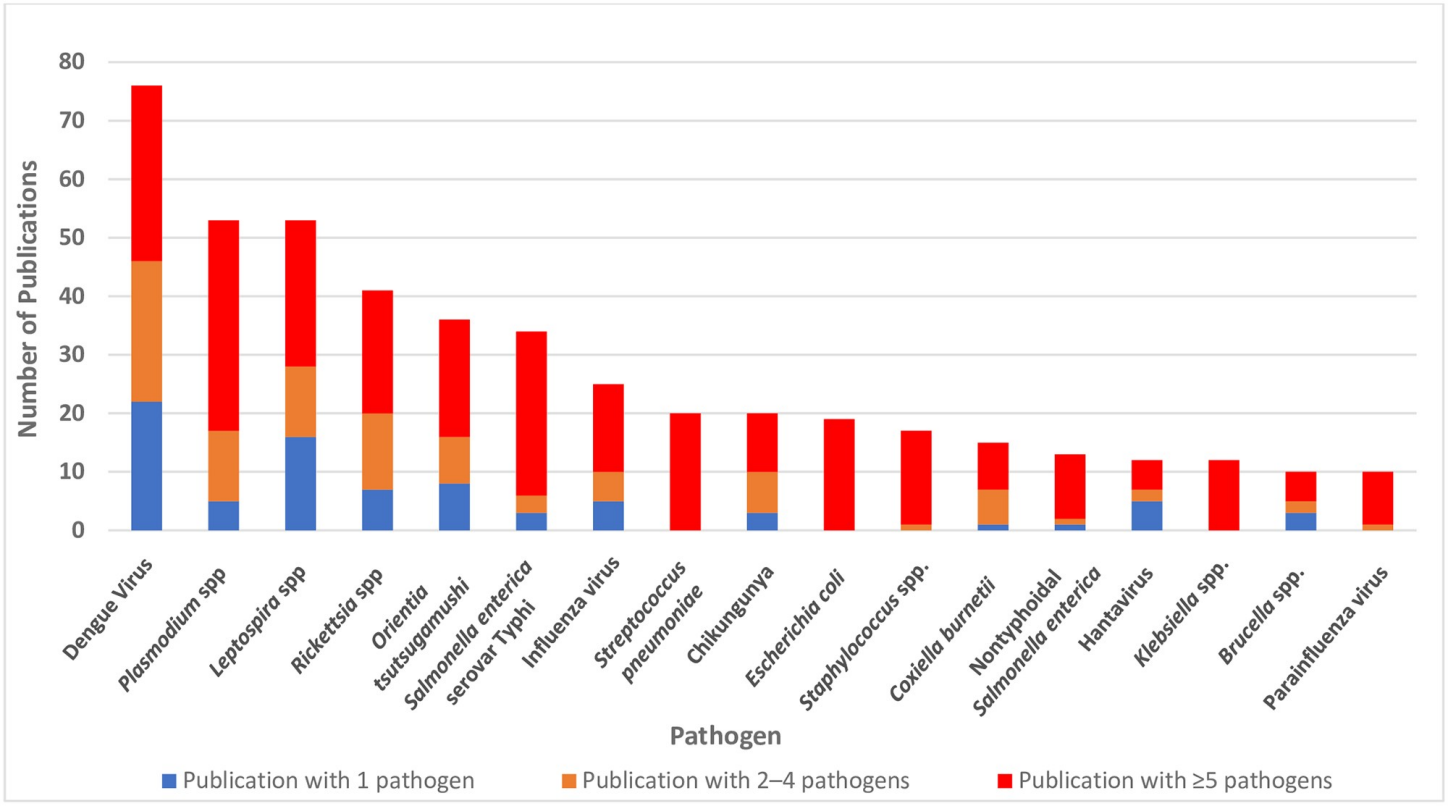
Number of publications by pathogen investigated as an etiology of acute febrile illness stratified by total number of investigated pathogens in the publication, from January 01, 2005 to December 31, 2017 (N = 190).

Pathogens that were included in at least ten publications are shown in Fig 3, with a complete list provided in S3 Table. Dengue virus, *Plasmodium* spp., and *Leptospira* spp. were the most commonly investigated or identified, with each represented in 76 (40%), 53 (28%), and 53 (28%) publications, respectively (S3 Table). The commonly investigated pathogens were represented in a mix of single-pathogen and multi-pathogen studies (Fig 3). Pathogen classification varied, with some studies defining pathogen at the genus level, while others defined it at the species, and sometimes serovar, level.

The use of laboratory methods for identification of pathogens that were included in at least ten publications, except for those that were primarily identified by blood culture, was also evaluated (Table 3). The proportion of publications using more than one diagnostic method or specimen type to test for a given pathogen ranged from two (14%) of 14 for *Coxiella burnetii* to five (56%) of nine for *Brucella* spp. The proportion of publications that used at least one standard laboratory diagnostic test (defined in S2 Table) to test for a given pathogen ranged from five of nine (56%) for *Brucella* spp. to 51 (100%) of 51 for *Plasmodium* spp.

**Table 3:** Diagnostic methods used to identify pathogens represented in ≥10 publications on etiologies of acute febrile illness, published from January 01, 2005 to December 31, 2017.

Pathogen	Total number of publications	Publications reported specific information on diagnostic method*	Publications by diagnostic methods	Publications with >1 diagnostic method (%)	Publications with standard† diagnostic method (%)
Dengue virus	76	74	NAAT: 33 Serology: 64 (PRNT 4) Culture: 3 Antigen testing: 20	39 (53%)	55 (74%)
*Plasmodium* spp.	53	51	NAAT: 7 Culture: 1 Microscopy: 44 Antigen testing: 19	15 (29%)	51 (100%)
*Leptospira* spp.	53	51	NAAT: 11 Serology: 46 (IFA 4, MAT 26) Culture: 7 Antigen testing: 4 Dark-field microscopy: 2	25 (49%)	34 (67%)
*Rickettsia* spp.	41	39	NAAT: 15 Serology: 32 (IFA 8)	12 (31%)	32 (82%)
*Orientia tsutsugamushi*	36	35	NAAT: 10 Serology: 34 (IFA 3) Culture: 2 Antigen testing: 1	12 (34%)	21 (60%)
*Salmonella enterica* serovar Typhi	34	34	NAAT: 3 Serology: 11 Culture: 24 Antigen testing:6	9 (26%)	24 (71%)
Influenza virus	25	23	NAAT: 21 Serology: 3 Antigen testing: 4	5 (22%)	21 (91%)
Chikungunya virus	20	20	NAAT: 12 Serology: 16 Culture: 1	9 (45%)	15 (75%)
*Coxiella burnetti*	15	14	NAAT: 1 Serology: 13	2 (14%)	12 (86%)
*Brucella* spp.	11	9	NAAT: 1 Serology: 9 Culture: 4	5 (56%)	5 (56%)

* Denominator for calculating proportions shown within the table

^†^ See S2 Table for US Centers for Disease Control and Prevention/Council of State and Territorial Epidemiologists (CDC/CSTE) or the World Health Organization (WHO) laboratory case definition by pathogen (abbreviation: NAAT = nucleic acid amplification test, PRNT = plaque reduction neutralization test, IFA = immunofluorescent antibody, MAT: microscopic agglutination test)

**NB**: Only pathogens that are included in ≥10 publications are shown

## Discussion

This scoping review of published AFI etiology investigation methods highlights several gaps that can be addressed for a more cohesive and impactful global AFI surveillance strategy. We found wide variation in the AFI case definitions, including the fever cut-off value, inclusion and exclusion criteria, and in how findings were reported across existing AFI publications. Use of laboratory diagnostics not meeting widely accepted laboratory case definitions was common. The lack of AFI investigation methods and reporting in publications assessed prevents data aggregation across geography and time that could meaningfully guide public health action.

Similar to existing reviews, we found gaps in the geographic distribution of study origin and in the demographics of the study populations. [6,7] There are a relatively large number of publications originating from the Southern Asia, Southeastern Asia, and Eastern Africa regions, in comparison to the Southern Africa, Northern Africa, and South America regions. Southern Asia, Southeastern Asia, and Eastern Africa are also regions of the world where malaria transmission is known to occur most frequently. [2] However, with a decline in malaria transmission and increased access to malaria diagnostics, it is important to identify non-malarial AFI etiologies and epidemiologic characteristics in these regions for future studies. [16] While we did not geographically limit our search based on country income classification, our search criteria resulted in publications with study settings largely within low- to middle-income countries. This is presumably driven by a greater need for these data in settings reliant on empiric AFI treatment guidance in the absence of widely available clinical diagnostic laboratory resources.

Among the AFI etiologies investigated, certain pathogens received a seemingly disproportionate amount of attention in the literature compared to others, for which a knowledge gap on their global distribution continues to persist. For example, dengue virus was discussed in 66 (39%) of reviewed publications. By contrast, fewer studies examined other pathogens, such as *Vibrio cholerae* (Fig 3, S3 Table). Several factors can influence the decision to study a pathogen, namely research interest, funding availability, diagnostic capacity, and existing evidence on the distribution of pathogens or competent vectors. As evidence of the information gaps commonly found with neglected pathogens, recently published literature on *O*. *tsutsugamushi* demonstrates a lack of epidemiologic evidence outside the traditionally endemic Asia-Pacific region despite the recent emergence of data suggesting autochthonous transmission of scrub typhus in South America and Africa. [17]

This review underscores a need for more comprehensive, multi-pathogen AFI etiologic studies paired with clinical presentation data. These etiologic investigations of AFI could be designed to unpack the circulating pathogens that contribute to AFI in geographic areas and quantify their attributable fractions, in order to ultimately guide empiric management and inform public health measures. Due to their specific and focused nature, single-pathogen studies provide limited evidence and data for actionable purposes or continue to reinforce pre-existing notions on the distribution of pathogens rather than document novel findings such as the presence of pathogens in new geographic areas or populations. Although there are many ongoing initiatives to characterize circulating causes of febrile illnesses, a paucity of evidence still prevails relative to that of etiologies responsible for respiratory and diarrheal diseases. [18,19]

Unlike influenza and malaria, where extensive global efforts have been implemented to build diagnostic capacity, laboratory confirmation remains challenging for many other AFI pathogens. Obtaining paired sera, the issue of cross-reactivity, and limited availability of assays that are considered confirmatory or the gold-standards (e.g., immunofluorescence assay (IFA) or microagglutination test (MAT)) add additional layers of difficulty in laboratory confirmation.

Strengthening laboratory diagnostic capacity for common pathogens responsible for AFI in low- and middle-income countries directly improves routine surveillance systems and availability of actionable data to guide public health interventions. The WHO Collaborating Center for International Health Regulations (IHR) Implementation of National Surveillance and Response Capacity recommends that countries develop their capacity to perform 10 core tests related to influenza, poliovirus, HIV, *Mycobacterium tuberculosis*, *Plasmodium* spp., *Salmonella enteritidis* serovar Typhi and four additional pathogens with local priority selected by each country. [20] As many of these agents can cause AFI, improving AFI surveillance can assist countries in developing and implementing core public health capacities as outlined in IHR, a fact which is readily apparent in practice. For example, the CDC’s Global Disease Detection (GDD) program has worked to strengthen AFI surveillance and laboratory capacity in low-resource countries, establishing platforms which have been used for investigating emerging pathogens such as Zika virus and *Orientia tsutsugamushi*. [21] More recently, experiences gained through sentinel laboratory-based arbovirus surveillance of AFI cases in Burkina Faso during a dengue outbreak were reported to be useful for building sustainable routine AFI surveillance. [22]

New technological advances in the field of laboratory diagnostics can certainly play a role in addressing the aforementioned challenges surrounding laboratory confirmation. [23–25] Increased utilization of both serological and NAAT-based multi-pathogen testing platforms has allowed AFI etiologic investigations to test for a broader set of pathogens including pathogens that might not have not been prioritized for testing if a purely single-pathogen testing strategy had been employed. [26–28] At the same time, increasing attention has been given to the development of point-of-care diagnostics and other easily-deployable laboratory diagnostics appropriate for the detection of non-malarial pathogens associated with AFI in low-resource settings. [29] There is also a potential role for advanced molecular detection–harnessing the combined capacities of next generation sequencing (NGS) and bioinformatics to more swiftly identify and characterize causes of disease–in very specific situations such as testing specimens without any positive results on standard testing or for fatal cases. [30] Technological advances in NGS have also allowed for rapid pathogen identification during outbreaks and pathogen discovery. [31–33] The current landscape with regards to these technological frontiers suggests that it may take substantial time and investment before these technologies achieve levels of performance, ease-of-use, and affordability required in order to become widely available and accessible across the globe.

These scoping review findings also highlight a lack of standardization in how methods and results from AFI studies are reported in the scientific literature. The need for standardized guidelines or even protocols for conducting AFI studies has previously been identified, and this lack of standardization continues to be a major impediment for being able to compare results from different studies and pool their results to develop national, regional or global burden of disease estimates. [6,7,34] For example, as shown in Table 2, the reported value for the fever cut-off ranged from <37·5°C to ≥38·5°C; because of such disparities, the prevalence data captured through each of these studies would vary from one study to the next. Standardization of AFI case definitions would enable more robust and consistent capture of AFI illness and pathogens, which would then allow more statistically sound comparisons of AFI etiologies between studies and across regions. Additionally, laboratory confirmation criteria for specific AFI etiologies, especially with the specific diagnostics employed to apply these case definitions, limit the ability to analyze AFI cause-specific trends between investigations. Standardized guidelines could also be useful for clarifying relatively neglected topics such as the role of control groups in estimating attributable fractions, or in setting quality recommended standards for studies to target. Unlike those in pneumonia and diarrhea, we were unable to identify a published multi-country etiologic investigation of AFI with standardized methodology other than one currently in progress. [18,35,36] Yet many valuable lessons can be learned from multi-country etiologic investigations on pneumonia and diarrhea, and the global AFI research community can benefit from many of their recommendations. [37,38]

To streamline AFI etiology reporting, minimize variation in key data elements, and improve our ability to fill existing knowledge gaps, we recommend the development and use of a checklist outlining specific criteria to be included in the methods and results sections of publications related to AFI studies in addition to use of existing checklists such as “Strengthening the Reporting of Observational Studies in Epidemiology” (STROBE) checklist. [39] A proposal of such a checklist specifically designed for AFI etiologic investigation can be found in Table 4. Such a checklist could be further developed by a wider group to become adopted as a standard framework for conducting AFI etiologic investigations. Complementing laboratory findings, future individual studies and framework consensus should consider the clinical sign and symptom co-variates needed to evaluate attribution, as well as the potential for these data to provide useful real-time public health alerts when monitored over time. Such data may help focus public health response to infectious disease outbreaks when additional diagnostic investigation for etiology is needed.

**Table 4:** Proposed reporting standard for studies on etiologic investigations of acute febrile illness, based on review of existing publications on AFI etiology, published from January 01, 2005 to December 31, 2017.

Section	[x]	Recommendation
Methods: Study design	[] [] [] [] [] []	Report the following: Study data collection methods Criteria for study site selection Study site locations Patient recruitment criteria Study duration Type of study (e.g., case-control, cross-sectional)
Methods: Setting	[] [] [] [] [] [] [] []	Describe catchment area characteristics relevant to AFI etiologies being investigated: Endemicity of known AFI etiologies in the region Geography Climate Precipitation Land use Urbanization Prevalence of underlying conditions, such as HIV, in similar reference population if not included in study itself
	[]	Indicate type of health care facility where participants are recruited
Methods: Participants	[] [] [] [] [] []	Provide detailed case definition: Fever cut-off value Site of body where measurement is taken Type of fever: subjective (self-reported, not measured at clinic) or measured at clinic Duration of fever Age/age groups Any other inclusion and exclusion criteria
	[] [] []	Provide the following if a control group is included: Identification and selection of controls Factors on which cases/controls were matched Number of controls
Methods: Laboratory	[] [] []	Laboratory case definition Criteria for determining a positive or detectable result Validation/verification of diagnostic method used, if not standard*
Results: Participants	[] [] []	Report total number of study participants enrolled Report study participant demographics Clinical signs and symptoms
Results: Laboratory data	[] []	Report total number of study participants tested for each pathogen/laboratory diagnostic method used Report total number of positive or detectable results for each pathogen/laboratory diagnostic method used
Results: Seasonal trends	[]	Describe seasonal trend, if applicable

*In alignment with widely accepted laboratory diagnostics and laboratory confirmation case definition guidelines preferably set forth by the CDC/Council of State and Territorial Epidemiologists (CSTE) or World Health Organization (WHO).

**NB**: This proposed standard should be refined through a consensus process prior to implementation. Once implemented, the finalized reporting standard could provide an ideal avenue for open data access and the ability to share data for aggregate analyses.

This scoping review is subject to a number of limitations. Literature assessed for this study was limited to that published in the English language from January 1, 2005–December 1, 2017. In addition, the literature was also limited to articles published in peer-reviewed journals meaning that gray literature, white papers, and routine surveillance reports were also omitted from consideration. As a result, there is a potential for bias in the analysis of gaps in geographic distributions of AFI etiologic investigations, as well as pathogens that have been studied. At the same time, data analysis to determine gaps in research was limited to information contained in articles, opening up the possibility of reporting bias. Along the same lines, the content and quality of each publication included in the review differed, lending themselves to variable interpretation from one reader to the next. It is possible, also, that differences in HIV prevalence among regions could impact study results and, therefore, publication focus; HIV-specific comorbidity risks should be considered in settings with high HIV prevalence.

### Conclusion

AFI etiologic investigations are not only important for shining a light on the causes of what is still a somewhat unexplored clinical presentation in many parts of the world but also for building surveillance and diagnostic capacity in countries where common causative pathogens are circulating. Looking ahead, technological advances such as the validation of accurate multi-pathogen testing platforms combined with improvements in the availability and accessibility of diagnostic tools, as well as increased testing for etiologies that are not well understood or commonly tested for can contribute to bridging the global AFI knowledge gaps. At the same time, there is great variability in AFI etiologic investigation methodologies, and the absence of standardized case definitions and reporting hinders our ability to generate a complete global picture of the causative agents of AFI. A coordinated and standardized methodology for reporting could help mitigate these inconsistencies and generate a more comprehensive understanding of AFI etiologies and distribution, potentially with measurable global health security and AFI survival benefit.

## Supporting information

S1 Table Search strategy used to determine the eligibility of publications on etiologies of acute febrile illness, published from January 01, 2005, to December 31, 2017 (N = 190). (DOCX)

S2 Table Standard laboratory case definition by pathogen, as defined by the US Centers for Disease Control and Prevention/Council of State and Territorial Epidemiologists (CDC/CSTE) or the World Health Organization (WHO). (DOCX)

S3 Table List of pathogens reported and number of publications by United Nations geographic regions published from published from January 01, 2005, to December 31, 2017, in which each pathogen was identified. (DOCX)

S1 File Data characterization forms for full-text review of publications on etiologies of acute febrile illness published from January 01, 2005 to December 31, 2017. (PDF)

S2 File Results of identified abstracts according to exclusion and inclusion criteria from publications on etiologies of acute febrile illness, published from January 01, 2005, to December 31, 2017. (XLSX)

S3 File (DOC)

S4 File (DOC)
