# Supplementary material for: Global knowledge gaps in acute febrile illness etiologic investigations: A scoping review
Source: PLoS Negl Trop Dis. 2019 Nov 15;13(11):e0007792. doi: 10.1371/journal.pntd.0007792 (PMC6881070; doi:10.1371/journal.pntd.0007792)
Supplement: S1 Table — (DOCX) [file pntd.0007792.s001.docx]

**S1 Table. Search strategy used to determine the eligibility of publications on etiologies of acute febrile illness, published from January 01, 2005 to December 31, 2017 (N=190)**

| **Database Search** | **Manual Search** |
| --- | --- |
| **Databases**   1. Embase 2. Global Health 3. Ovid MEDLINE | Mapping the aetiology of non-malarial febrile illness in Africa and SE Asia Project (Available at:  <http://www.wwarn.org/non-malarial-febrile-illness-nmfi-map>) |
| **Search terms and search strategy**   1. (undifferentiated adj2 fever).ab. 2. (undifferentiated adj2 fever).ti. 3. (acute adj2 febrile adj2 illness).ab. 4. (acute adj2 febrile adj2 illness).ti. 5. (non$specific adj2 fever).ab. 6. (non$specific adj2 fever).ti. 7. 1 or 2 or 3 or 4 or 5 or 6 8. Limit 7 to abstracts 9. Limit 8 to English language 10. Limit 9 to human 11. Journal article.pt. 12. 10 and 11 13. Limit 12 to yr=”2005-2017” 14. Remove duplicates | **Other review publications**   1. Acestor N, Cooksey R, Newton PN, et al. Mapping the aetiology of non-malarial febrile illness in Southeast Asia through a systematic review--terra incognita impairing treatment policies. PLoS One. 2012;7(9):e44269. 2. Prasad N, Murdoch DR, Reyburn H, Crump JA. Etiology of Severe Febrile Illness in Low- and Middle-Income Countries: A Systematic Review.PLoS One. 2015;10(6):e0127962 3. Reddy EA, Shaw AV, Crump JA. Community-acquired bloodstream infections in Africa: a systematic review and meta-analysis. Lancet Infect Dis. 2010;10(6):417-32. 4. Deen J, von Seidlein L, Andersen F, et al. Community-acquired bacterial bloodstream infections in developing countries in south and southeast Asia: a systematic review. Lancet Infect Dis. 2012;12(6):480-7 5. Church J, Maitland K. Invasive bacterial co-infection in African children with Plasmodium falciparum malaria: a systematic review. BMC Med. 2014;12:31. doi: 10.1186/1741-7015-12-31. |
| **Search result:** 1,083 Abstracts | **Search result:** 256 abstracts |
