# Supplementary material for: Global knowledge gaps in acute febrile illness etiologic investigations: A scoping review
Source: PLoS Negl Trop Dis. 2019 Nov 15;13(11):e0007792. doi: 10.1371/journal.pntd.0007792 (PMC6881070; doi:10.1371/journal.pntd.0007792)
Supplement: S2 Table — (DOCX) [file pntd.0007792.s002.docx]

**S2 Table. Standard laboratory case definition by pathogen, as defined by the US Centers for Disease Control and Prevention/Council of State and Territorial Epidemiologists (CDC/CSTE) or the World Health Organization (WHO)**

| **Pathogen** | **Standard Case Definition** | | | |
| --- | --- | --- | --- | --- |
|  | **Culture** | **Serology** | **NAAT** | **Others (Microscopy, Antigen, Pathology, etc.)** |
| Dengue virus | Viral culture and Isolation from serum, plasma, or CSF | IgM anti-DENV seroconversion by immunoassay in acute and convalescent, IgG anti-DENV seroconversion; **OR** ≥4-fold rise in titer in serum specimens collected >2 weeks apart, and confirmed by a neutralization test with a >4-fold higher end point titer as compared to other flaviviruses tested | Detection of DENV nucleic acid in serum, plasma, blood, cerebrospinal fluid (CSF), other body fluid or tissue | Detection in serum or plasma of DENV NS1 antigen; **OR** Detection of DENV antigens in tissue by immunofluorescence or immunohistochemistry assay |
| Chikungunya virus | Virus isolation from blood in the first few days of illness | Four-fold or greater change in virus-specific quantitative antibody titers in paired sera; **OR** virus specific IgM antibodies in serum with confirmatory virus-specific neutralizing antibodies in the same or a later specimen; **OR** virus-specific IgM antibodies in CSF or serum | Demonstration of nucleic acid in tissue, blood, CSF or other body fluid | Demonstration of specific viral antigen in tissue, blood, CSF, or other body fluid |
| *Plasmodium* spp (malaria) |  |  | Detection of *Plasmodium* spp by nucleic acid test | Malaria rapid diagnostic test; **OR**  Detection and specific identification of malaria parasite species by microscopy on blood films |
| *Rickettsia* spp | Isolation of *Rickettsia* spp from a clinical specimen in cell culture | 4-fold increase in immunoglobulin G (IgG)-specific antibody titer by indirect immunofluorescence assay between acute and convalescent phase specimens | Detection of DNA in a clinical specimen via amplification of a specific target | Demonstration of antigen in a biopsy or autopsy specimen by IHC |
| *Orientia tsutsugamushi* | Culture and Isolation | 4-fold increase in immunoglobulin specific antibody titer by indirect immunofluorescence assay between acute and convalescent phase specimens | Detection of DNA in a whole blood, eschar swab, or tissue sample via amplification of a specific target | Immunostaining on formalin-fixed tissue samples |
| Acute *Coxiella burnetii* | Isolation of from a clinical specimen by culture | 4-fold increase in immunoglobulin G specific antibody titer by indirect immunofluorescence assay between acute and convalescent phase specimens | Detection of DNA in a clinical specimen via amplification of a specific target | Demonstration in a clinical specimen by immunohistochemical methods |
| *Salmonella enterica* serovar Typhi | Isolation of *S.* Typhi from blood, stool, or other clinical specimen |  |  |  |
| *Leptospira* spp. | Isolation from a clinical specimen | 4-fold increase in *Leptospira* agglutination titer between acute and convalescent phase specimens, *Leptospira* agglutination titer of ≥ 800 by Microscopic Agglutination Test (MAT) | Detection of pathogenic *Leptospira* DNA from a clinical specimen | Demonstration a in tissue by direct immunofluorescence |
| *Brucella* spp. | Culture and identification from clinical specimens | 4-fold increase in *Brucella* antibody titer between acute and convalescent phase specimens; **OR** Coombs IgG |  |  |
| *Mycobacterium tuberculosis* | Isolation of *M. tuberculosis* from a clinical specimen |  | Demonstration of *M. tuberculosis* complex from a clinical specimen by nucleic acid amplification test | Demonstration of acid-fast bacilli in a clinical specimen |
| Influenza virus | Influenza virus isolation in tissue cell culture from respiratory specimens | Immunofluorescent antibody staining (direct or indirect) of respiratory specimens OR Four-fold rise in influenza hemagglutination inhibition (HI) antibody titer in paired acute and convalescent sera | Reverse-transcriptase polymerase chain reaction (RT-PCR) testing of respiratory specimens | Immunohistochemical (IHC) staining for influenza viral antigens in respiratory tract tissue from autopsy specimens OR Rapid influenza diagnostic testing of respiratory specimens |
