# Supplementary material for: Global knowledge gaps in acute febrile illness etiologic investigations: A scoping review
Source: PLoS Negl Trop Dis. 2019 Nov 15;13(11):e0007792. doi: 10.1371/journal.pntd.0007792 (PMC6881070; doi:10.1371/journal.pntd.0007792)
Supplement: S3 Table — (DOCX) [file pntd.0007792.s003.docx]

**S3 Table. List of pathogens reported and number of publications by United Nations geographic regions published from January 01, 2005 to December 31, 2017, in which each pathogen was identified**

| **Pathogen** | **Africa** | **Americas** | **Asia** | **Europe** | **Oceania** | **Total** |
| --- | --- | --- | --- | --- | --- | --- |
| Dengue virus | 8 | 16 | 49 | 1 | 2 | 76 |
| *Plasmodium* spp. | 19 | 3 | 27 | 1 | 3 | 53 |
| *Leptospira* spp. | 4 | 9 | 38 | 0 | 2 | 53 |
| *Rickettsia* spp. | 11 | 4 | 25 | 0 | 1 | 41 |
| *Orientia tsutsugamushi* | 0 | 0 | 35 | 0 | 1 | 36 |
| *Salmonella enterica* serovar Typhi | 13 | 0 | 21 | 0 | 0 | 34 |
| Influenza virus | 6 | 6 | 11 | 0 | 2 | 25 |
| *Streptococcus pneumoniae* | 14 | 1 | 3 | 0 | 2 | 20 |
| Chikungunya virus | 7 | 2 | 11 | 0 | 0 | 20 |
| *Escherichia coli* | 12 | 0 | 5 | 0 | 2 | 19 |
| *Staphylococcus* spp. | 11 | 0 | 4 | 0 | 2 | 17 |
| *Coxiella burnetii* | 4 | 4 | 5 | 1 | 1 | 15 |
| Nontyphoidal *Salmonella enterica* | 11 | 0 | 1 | 1 | 0 | 13 |
| Hantavirus | 2 | 1 | 9 | 0 | 0 | 12 |
| *Klebsiella* spp. | 8 | 0 | 3 | 0 | 1 | 12 |
| *Brucella* spp. | 5 | 1 | 4 | 0 | 0 | 11 |
| Parainfluenza virus | 3 | 3 | 4 | 0 | 0 | 10 |
| Hepatitis A virus | 1 | 0 | 7 | 1 | 0 | 9 |
| Japanese Encephalitis virus | 0 | 0 | 8 | 0 | 1 | 9 |
| *Haemophilus influenzae* | 6 | 0 | 1 | 0 | 1 | 8 |
| *Mycobacterium tuberculosis* | 3 | 0 | 4 | 1 | 0 | 8 |
| Adenovirus | 3 | 3 | 1 | 0 | 1 | 8 |
| Respiratory Syncytial virus | 3 | 2 | 2 | 0 | 1 | 8 |
| *Bartonella* spp. | 1 | 0 | 5 | 1 | 0 | 7 |
| West Nile virus | 4 | 1 | 2 | 0 | 0 | 7 |
| Enterovirus | 2 | 4 | 1 | 0 | 0 | 7 |
| Ebstein-Barr virus | 1 | 1 | 2 | 0 | 2 | 6 |
| *Enterobacter* spp. | 4 | 0 | 2 | 0 | 0 | 6 |
| *Burkholderia* spp. | 0 | 1 | 3 | 0 | 2 | 6 |
| Group A *Streptococcus* | 6 | 0 | 0 | 0 | 0 | 6 |
| *Pseudomonas* spp. | 4 | 0 | 2 | 0 | 0 | 6 |
| *Cryptococcus* spp. | 3 | 0 | 2 | 0 | 0 | 5 |
| HIV | 2 | 0 | 2 | 1 | 0 | 5 |
| Yellow Fever virus | 3 | 2 | 0 | 0 | 0 | 5 |
| Human Metapneumovirus | 3 | 2 | 0 | 0 | 0 | 5 |
| Coronavirus | 2 | 3 | 0 | 0 | 0 | 5 |
| Hepatitis B virus | 0 | 0 | 5 | 0 | 0 | 5 |
| *Acinetobacter* spp. | 2 | 0 | 2 | 0 | 0 | 4 |
| Crimean-Congo Hemorrhagic Fever Virus | 1 | 0 | 2 | 1 | 0 | 4 |
| Cytomegalovirus | 0 | 1 | 2 | 0 | 1 | 4 |
| *Neisseria meningitidis* | 1 | 1 | 1 | 0 | 1 | 4 |
| Mayaro virus | 0 | 3 | 0 | 1 | 0 | 4 |
| *Mycoplasma* spp. | 2 | 0 | 1 | 0 | 1 | 4 |
| Rhinovirus | 2 | 2 | 0 | 0 | 0 | 4 |
| Hepatitis E virus | 1 | 0 | 3 | 0 | 0 | 4 |
| *Borellia* spp. | 3 | 0 | 0 | 0 | 0 | 3 |
| *Citrobacter* spp. | 2 | 0 | 1 | 0 | 0 | 3 |
| Phlebovirus | 1 | 0 | 2 | 0 | 0 | 3 |
| *Proteus* spp. | 2 | 0 | 0 | 0 | 1 | 3 |
| Varicella Zoster virus | 0 | 0 | 2 | 0 | 1 | 3 |
| *Anaplasma phagocytophilum* | 1 | 1 | 1 | 0 | 0 | 3 |
| Lassa Fever virus | 3 | 0 | 0 | 0 | 0 | 3 |
| Oropouche virus | 0 | 3 | 0 | 0 | 0 | 3 |
| Zika virus | 1 | 1 | 1 | 0 | 0 | 3 |
| Bocavirus | 1 | 1 | 0 | 0 | 0 | 2 |
| Herpes simplex virus | 0 | 1 | 1 | 0 | 0 | 2 |
| *Histoplasma* spp. | 2 | 0 | 0 | 0 | 0 | 2 |
| Orthobunyavirus | 0 | 2 | 0 | 0 | 0 | 2 |
| Parechovirus | 1 | 1 | 0 | 0 | 0 | 2 |
| Parvovirus B19 | 0 | 1 | 0 | 1 | 0 | 2 |
| Rubella virus | 0 | 1 | 1 | 0 | 0 | 2 |
| Tick-Borne Encephalitis virus | 0 | 0 | 2 | 0 | 0 | 2 |
| Venezuelan Equine Encephalitis virus | 0 | 2 | 0 | 0 | 0 | 2 |
| Viridans *streptococci* | 1 | 0 | 1 | 0 | 0 | 2 |
| *Bordetella pertussis* | 2 | 0 | 0 | 0 | 0 | 2 |
| *Chlamydophila pneumoniae* | 1 | 0 | 1 | 0 | 0 | 2 |
| *Moraxella* spp. | 2 | 0 | 0 | 0 | 0 | 2 |
| Astrovirus | 1 | 0 | 0 | 0 | 0 | 1 |
| *Bacteroides* spp. | 1 | 0 | 0 | 0 | 0 | 1 |
| BK virus | 0 | 1 | 0 | 0 | 0 | 1 |
| Bunyavirus | 0 | 0 | 1 | 0 | 0 | 1 |
| *Campylobacter* spp. | 1 | 0 | 0 | 0 | 0 | 1 |
| *Clostridium difficile* | 0 | 0 | 0 | 1 | 0 | 1 |
| *Corynebacteria* spp. | 1 | 0 | 0 | 0 | 0 | 1 |
| *Cryptosporidium* spp. | 1 | 0 | 0 | 0 | 0 | 1 |
| Dobrova virus | 0 | 0 | 0 | 1 | 0 | 1 |
| Group B *Streptococcus* | 1 | 0 | 0 | 0 | 0 | 1 |
| Group C *Streptococcus* | 1 | 0 | 0 | 0 | 0 | 1 |
| Guaroa virus | 0 | 1 | 0 | 0 | 0 | 1 |
| Ilheus Virus | 0 | 1 | 0 | 0 | 0 | 1 |
| KI Virus | 0 | 1 | 0 | 0 | 0 | 1 |
| *Leishmania* spp. | 0 | 0 | 1 | 0 | 0 | 1 |
| *Micrococcus* spp. | 0 | 0 | 1 | 0 | 0 | 1 |
| Morbilli virus | 1 | 0 | 0 | 0 | 0 | 1 |
| Mumps virus | 0 | 0 | 0 | 0 | 1 | 1 |
| Norovirus | 1 | 0 | 0 | 0 | 0 | 1 |
| *Pantoea* spp. | 0 | 0 | 1 | 0 | 0 | 1 |
| Picornavirus | 0 | 1 | 0 | 0 | 0 | 1 |
| *Pneumocystis jiroveci* | 0 | 0 | 0 | 1 | 0 | 1 |
| *Providencia* spp. | 1 | 0 | 0 | 0 | 0 | 1 |
| Puumala virus | 0 | 0 | 0 | 1 | 0 | 1 |
| Rift Valley Fever virus | 1 | 0 | 0 | 0 | 0 | 1 |
| Rotavirus | 1 | 0 | 0 | 0 | 0 | 1 |
| Sapovirus | 1 | 0 | 0 | 0 | 0 | 1 |
| *Serratia* spp. | 1 | 0 | 0 | 0 | 0 | 1 |
| *Shigella* spp. | 1 | 0 | 0 | 0 | 0 | 1 |
| St Louis Encephalitis virus | 0 | 1 | 0 | 0 | 0 | 1 |
| Tahyna virus | 1 | 0 | 0 | 0 | 0 | 1 |
| Tonate virus | 0 | 0 | 0 | 1 | 0 | 1 |
| Toxoplasma gondii | 0 | 0 | 0 | 1 | 0 | 1 |
| *Vibrio cholerae* | 1 | 0 | 0 | 0 | 0 | 1 |
| Wu virus | 0 | 1 | 0 | 0 | 0 | 1 |
| *Wuchereria bancrofti*, *Brugia malayi* | 0 | 0 | 1 | 0 | 0 | 1 |
| *Legionella* spp. | 0 | 0 | 1 | 0 | 0 | 1 |
| Hepatitis C virus | 0 | 0 | 1 | 0 | 0 | 1 |
