# Supplementary material for: Global knowledge gaps in acute febrile illness etiologic investigations: A scoping review
Source: PLoS Negl Trop Dis. 2019 Nov 15;13(11):e0007792. doi: 10.1371/journal.pntd.0007792 (PMC6881070; doi:10.1371/journal.pntd.0007792)
Supplement: S1 File — (PDF) [file pntd.0007792.s004.pdf]

AFI Scoping Review Full Text Data Collection Form Version 1.0

Part A: General

Study ID (as assigned)

Name of Reviewer (initials)

Year of Study Publication (YYYY)

Year Study Started (YYYY)

Month of Study Start (If Applicable)

Year Study Ended (YYYY)

Month of Study End (If Applicable)

Part B: Study Setting, Funding, and Researchers

Number of Countries Study Conducted

Sub-national level (Region/Province/City, list all if multiple)

Country Name

Name of country 1

Name of country 2

Name of country 3

Name of country 4

Name of country 5

Name of country 6

Malaria Endemic (if article specifically mentions)

Dengue Endemic (if article specifically mentions)

Study Population Setting (per mention in article)

- Urban
- Rural
- Both
- Not specified

Setting of Patient Encounter

- Inpatient Only
- Ambulatory/Emergency Only
- Community Only
- Mixed
- Not Defined

Type of funding organization (Check all that apply)

- [ ] Academia
- [ ] Foreign Government
- [ ] Local Government
- [ ] Foundation, NGO, Charity
- [ ] Corporate, Pharma, Biotech
- [ ] Multilateral organization (e.g. WHO)

Type of author affiliation (Check all that apply)

- [ ] Academia
- [ ] Foreign Government
- [ ] Local Government
- [ ] Foundation, NGO, Charity
- [ ] Corporate, Pharma, Biotech
- [ ] Multilateral organization (e.g. WHO)

Name of funding organization(s) (e.g., CDC, Duke University, Gates Foundation, etc.)

Name of author organization(s) (e.g., CDC, Duke University, Ministry of Health, etc.)

Part C: Design

Study Design

- ○ Case Series
- ○ Cross-Sectional
- ○ Cohort
- ○ Case-Controlled
- ○ Interventional
- ○ Surveillance
- ○ Other

Study Design Other (briefly explain)

Control Group

- ○ Yes
- ○ No
- ○ Not Applicable

Study Design Temporality

- ○ Cross-Sectional
- ○ Prospective
- ○ Retrospective
- ○ Unspecified

How control was selected? (briefly explain)

Population Under Surveillance Known (specifically mentioned or can be inferred)

- ○ Yes
- ○ No
- ○ Not applicable
- ○ Not mentioned and can't be inferred

Study Age Range (Check all that apply if study age range even partically included)

- ☐ Neonate (<1m)
- ☐ Infant (1m-23m)
- ☐ Children (2-9y)
- ☐ Adolescents (10-19y)
- ☐ Young Adult (20-39y)
- ☐ Middle-Aged Adult (40-59y)
- ☐ Elderly (>=60y)

Was any type of seasonality reported? (figure or table or mentioned in text)

○ Yes ○ No

How seasonality reported (briefly explain including pathogens)

Primary Study Objective (Check the most appropriate one)

- ○ Epi assessment of etiology – species level (e.g. Plasmodium falciparum)
- ○ Epi assessment of etiology – genus level (e.g. Rickettsia spp.)
- ○ To broadly define AFI etiology (e.g. multiple species/genus)
- ○ Describe Clinical Scenario
- ○ Study Diagnostic Test
- ○ Assess Intervention
- ○ Detection of Novel or Emerging Pathogen
- ○ Other or Uncertain (Please explain in detail)

Other Primary Study Objective

Secondary Study Objectives (Check all that apply, both mentioned or inferred)

- ☐ Epi assessment of etiology – species level
- ☐ Epi assessment of etiology – genus level
- ☐ To broadly define AFI etiology
- ☐ Describe Clinical Scenario
- ☐ Study Diagnostic Test
- ☐ Assess Intervention
- ☐ Other or Uncertain (Please explain in detail)

Other Secondary Study Objective

Part D: AFI Definition

AFI terminology

Terminology to describe acute fever in the study

If other, please specify

Measured Fever

Axillary Fever Cutoff

Temporal Fever Cutoff

Tympanic Fever Cutoff

Oral Fever Cutoff

Rectal Fever Cutoff

Unspecified Location Cutoff

Subjective Fever

Use of Tactile Fever

- Yes
- No
- Not specified

Subjective Fever

Number of days specified

- Specified
- Not specified

If specified, write number of days

Exclusion Criteria

Medical History

- Upper Limit of Fever Duration
- Previously Diagnosed Cause
- Recent Antibiotic Use
- Other (Specify)

Symptoms

- Upper/Lower Respiratory Symptoms
- Skin/Soft Tissue Infection
- STI Symptoms
- Gastrointestinal Symptoms
- CNS Symptoms
- Other (Specify)
- Urinary Tract Symptoms
- Dengue Hemorrhagic Fever Symptoms

Past Medical History

- HIV
- Renal Failure
- Recent/Planned Surgery
- Other (Specify)
- Malignancy
- Hepatic Failure
- Trauma

Lab Tests

- Lab Value (Specify)
- Malaria Positive
- Other Pathogen Test (Specify)

Other Exclusion Criteria

Describe "other"

Inclusion Criteria

Past Medical History

- Fever Longer Than Specific Interval
- HIV
- Renal Failure
- Other (Specify)

Lab Tests

- Lab Value (Specify)
- Malaria Positive
- MalariaNegative
- Other Pathogen Test (Specify)

Other Specific Inclusion

Free Text Inclusion

Part E: Overall Study Results and Diagnostics

Study Overall Results

Number of AFI cases enrolled

Number of controls enrolled (if applicable)

Number Pathogens Tested except by culture (for AFI)

Number Pathogens Tested except by culture (As control to AFI)

Total number of AFI cases diagnosed (per article)

Total number of controls diagnosed (per article)

Percentage AFI Cases Defined (diagnosed)

Percentage Controls Defined (If Applicable)

Was co-infection reported?

Multi-pathogen Laboratory Platform

☐ Blood Culture Algorithm

Blood Culture System

Culture Speciation

Other information regarding blood culture

☐ Respiratory Viral Panel

Panel Name

Respiratory Panel Specimen type

☐ Stool Enteric Panel

Enteric Panel Name

☐ Blood PCR

Blood PCR Name

PCR Specimen Type

Other platform

Total Number of Pathogens Identified in the Study? (Consider sub-species as one except Samonella typhi and non-typhoidal)

Please have the exact number of pathogens available before answering next section.

Instructions

- Please complete this for every pathogen.
- If there is only one diagnostic modality used for a pathogen, please just fill out for one.
- Check "confirmatory test" only when the study used confirmatory diagnostics for the pathogen
- Please use Comments section for any other information. (Assay, etc.)
- Use only numeric values for total specimens tested and total specimens tested positive. (Not proportions)

Pathogen

Name of Pathogen

Other Pathogen Specify

Test Modality 1

Sample Type 1

Test Modality 1 Product Name (If Applicable)

Total Specimens Tested 1

Total Specimens Tested Positive 1

☐ Confirmatory Test

Test Modality 2

Sample Type 2

Test Modality 2 Product Name (If Applicable)

Total Specimens Tested 2

Total Specimens Tested Positive 2

☐ Confirmatory Test

Test Modality 3

Sample Type 3

Test Modality 3 Product Name (If Applicable)

Total Specimens Tested 3

Total Specimens Tested Positive 3

☐ Confirmatory Test

Convalescent samples tested (If Applicable)

Comments (Please record anything significant that were left out for this pathogen)
