# Supplementary material for: Global knowledge gaps in acute febrile illness etiologic investigations: A scoping review
Source: PLoS Negl Trop Dis. 2019 Nov 15;13(11):e0007792. doi: 10.1371/journal.pntd.0007792 (PMC6881070; doi:10.1371/journal.pntd.0007792)
Supplement: S4 File — (DOC) [file pntd.0007792.s007.doc]

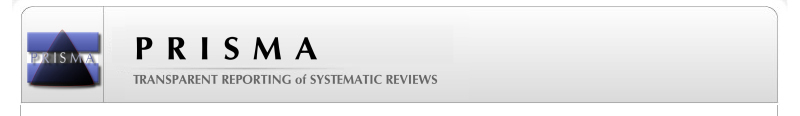
**PRISMA 2009 Flow Diagram**

**Screening**

**Included**

**Eligibility**

**Identification**

Records identified through database searching
(n = 1,083)

Additional records identified through other sources
(n = 256)

Records after duplicates removed
(n = 1,137)

Records screened
(n = 1,137)

Records excluded
(n = 947)

Full-text articles assessed for eligibility
(n = 190)

Full-text articles excluded, with reasons
(n = 0)

Studies included in qualitative synthesis
(n = 190)

Studies included in quantitative synthesis (meta-analysis)
(n = 190)
